# The Role of Household Structure and Composition in Influencing Complementary Feeding Practices in Ethiopia

**DOI:** 10.3390/nu14010130

**Published:** 2021-12-28

**Authors:** Asnake Ararsa Irenso, Dan Chamberlain, Miaobing Zheng, Karen J. Campbell, Rachel Laws

**Affiliations:** 1 School of Public Health, Haramaya University, Harar P.O. Box 235, Ethiopia; 2 Institute for Physical Activity and Nutrition (IPAN), School of Nutrition and Exercise Sciences, Deakin University, 221 Burwood Highway, Burwood, VIC 3125, Australia; j.zheng@deakin.edu.au (M.Z.); karen.campbell@deakin.edu.au (K.J.C.); r.laws@deakin.edu.au (R.L.); 3 Centre for Social Impact, University of New South Wales, Kennington, NSW 2052, Australia; d.chamberlain@unsw.edu.au

**Keywords:** household structure, Minimum Dietary Diversity, Minimum Meal Frequency, household composition, Ethiopia

## Abstract

While the household in which a child grows up is considered a critical environment that influences nutrition outcomes, there is little research examining the influence of household composition and structure on complementary feeding practices. This study examined the influence of household structure and composition on complementary feeding practices, using the Ethiopian Demographic and Health Survey (EDHS), 2000 to 2016. The composition variables were calculated from the attributes of household members (alters) and the structure variables from their kinship status. A multilevel mixed-effects regression model, specifying survey rounds as the random effect, was used to examine the association between household structure/composition and the Minimum Meal Frequency (MMF) and Minimum Dietary Diversity (MDD). The average Marginal Effects (MEs) were calculated to facilitate practical interpretation. Children of caregivers with a higher number of alters (degree), unique number of kinship category (effect size), closely related (constraint), and mixed-age alters (age diversity) seemed to increase the probability of meeting the MDD. Degree and effective size decreased the probability of meeting MMF, while constraint increased it. Overall, this study revealed some associations between household structure and composition and complementary feeding practices. Hence, complementary feeding interventions could be adapted to account for the household structure and composition variations.

## 1. Introduction

Optimal nutrition during the first 1000 days of life, spanning from conception to age two, lays the foundation for child health and well-being [1]. Early life nutrition is of key importance in Ethiopia, with child malnutrition being highly prevalent, with 2017 data reporting stunting at 42.0% (95% CI: 37.0, 46.0), underweight at 33.0% (95% CI: 27.0, 39.0), and wasting at 15.0% (95% CI: 12.0, 19.0) [2]. Malnutrition is predicted by multiple factors, including poor complementary feeding practices [3].

Suboptimal complementary feeding is defined by poor adherence to optimal feeding recommendations, such as the appropriate timing of introducing foods at around six months of age, providing a diverse diet with adequate meal frequency, and continued breastfeeding up to two years of age [4]. A recent systematic review on the quality of complementary feeding practices in Ethiopia highlighted that around 18% of children met Minimum Dietary Diversity (MDD) requirements (defined as eating at least four food groups per day), and 56% of children met the Minimum Meal Frequency (MMF) (consuming a recommended minimum number of times for their age) [5].

Complementary feeding practices are influenced by longstanding child-feeding traditions and social norms within families [6]. These traditions and norms are embedded in the household interpersonal/social environment, and they determine who feeds the child, the timing of introduction of foods, and what is fed and how. The household, a unit of interaction among the people sharing a living space irrespective of their relationship, is a child’s and their caregiver’s primary social field [7], and as such, it is an important focus for providing targeted support to caregivers [8]. Household members direct and indirect actions influence child taste preferences, eating habits and nutritional outcomes [6,9,10,11].

A rapid transformation in household structure has occurred worldwide, with consequences for broader social changes and nutrition, and the associated policy implication is a priority research agenda for African demographers [12]. The African Demographic and Health Survey between 1990 and 2011 showed that the predominant extended family forms, consisting of at least three generations, namely grandparents, married offspring, and grandchildren, have gradually declined with a concurrent increase in two-parent nuclear families. Evidence is scarce on country-specific family structures, which is essential to inform family-focused policies [13].

There is some evidence that household structure and composition can influence health and growth outcomes [14]. The household structure denotes a sociological dimension that emanates from marriage, childbearing, family living arrangements, and working members [15]. A study in Ghana showed that the nuclear family type had better health outcomes than extended-family households [16]. Similarly, in Botswana, children who live with a non-nucleated family had a higher risk of stunting [17].

Household composition variations and influences on child-feeding practices have not been examined before. It is important to understand differences in mothers’ responsibilities with complementary feeding practices compared to someone else delegated on her behalf to care for her child. Moreover, the child co-residence arrangements and household members’ similarities and diversities might determine the social support provided to the child’s primary caregiver, including supports for complementary feeding. Hence, this study aimed to provide an in-depth analysis of the Ethiopian household structure and composition and associated influences on complementary feeding practices, using four waves of the Ethiopian Demographic and Health Survey (EDHS) between 2000 to 2016. Associations by survey rounds and the combined effect of all survey rounds were evaluated to inform policy and practice recommendations in Ethiopia.

## 2. Materials and Methods

### 2.1. Study Design

This is a secondary-data analysis of the four Ethiopian Demographic and Health Survey (EDHS), a nationally representative survey conducted every five years in Ethiopia. EDHS was conducted in 2000 (February–June 2000) [14], 2005 (April–August 2005) [15], 2011 (December–June 2011) [16], and 2016 (January–June 2016) [17]. The survey was conducted in nine geographic regions and two city administrations, which vary in population size. Hence, the representativeness was insured by applying the sampling weight.

### 2.2. Study Population

The EDHS survey participants were selected in two stages. First, the enumeration areas (EAs), geographic areas, were chosen, followed by selecting households. The household data were collected from any adult member capable of providing information for usual household members and visitors on sex, age, relationship to the head of the household, education, parental survivorship, and residence [18]. In addition, a household members list was used to help identify eligible women and children under five years of age living in selected households.

### 2.3. Populations of Analysis

Eligible households were those with children between 6 and 24 months of age and where the women completed the infant-feeding-practices questionnaire (Rutstein, Rojas et al. 2006).

### 2.4. Variables

The social-network variables were calculated with EgoNet [19]. The variables were calculated from alters demographic characteristics and relationships among them, estimated by their kinship status. Alters are household members identified by their relationship to the women respondents, e.g., daughter or son, daughter- or son-in-law, grandchild, mother or father, parent-in-law, sister or brother, adopted/foster/stepchild, another relative, niece or nephew, and unspecified and non-relatives. These categories allow the identification of relationships by blood (such as daughter or son, mother or father, grandchild, sister or brother, another relative, and niece or nephew), by marriage (e.g., husband and in-laws) and those that are unrelated (e.g., non-relatives and adopted/foster/stepchild).

**Figure 1:**
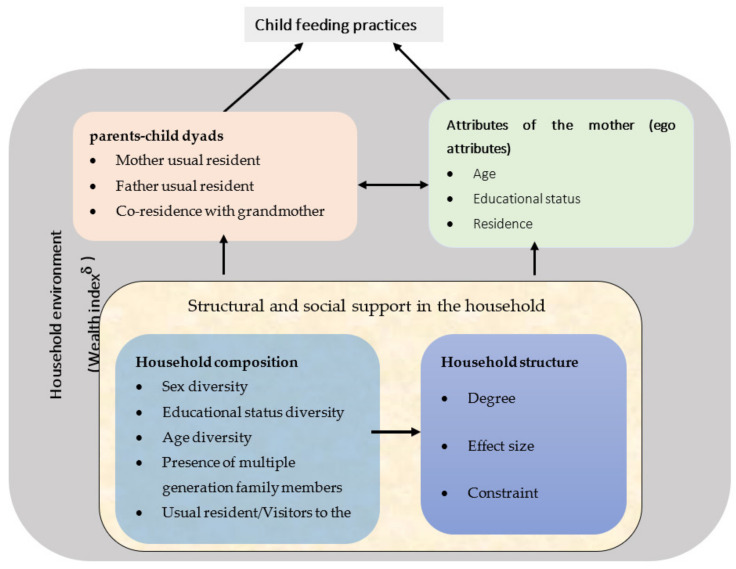
Modified conceptual framework of the influence of the household structure and composition on the feeding practices among children above six months of age [20,21]. ^δ^ The wealth index is a covariate related to the broader household environment, not specific to any members.

According to kin selection theory, closely related people have better interaction, cooperation, and altruism, influencing parental investment in their children, including feeding practices (L. Hamilton, Cheng, and Powell 2007; W.D. Hamilton 1964; Kuranchie 2021). In the Ethiopian context, typical rural households have three generations, while the urban households predominantly have a nuclear family structure (Evason 2018). The selection of variables was guided by a conceptual framework of feeding practices among children above six months of age (Figure 1) [20,21], as outlined below.

#### 2.4.1. Respondent (Ego) Attributes

The woman respondent is assumed as the center of attention and has a tie with all household members, and is called ego, which can be maternal or non-maternal. Women attributes considered in the analysis include age in years, educational level (no education, primary education and above), residence (urban/rural), type of earnings (working but not paid, paid in cash or in-kind or both, and not working) and wealth index. The wealth index was a precalculated variable of EDHS constructed from a long list of household items possessions, categorized into low, middle, and high terciles. The respondent and household characteristics measurement scale and analysis plan are summarised in Supplementary File S1.

#### 2.4.2. Household Composition

A household is a person or a group of persons who usually live and eat together and may consist of related and unrelated people. A household differs from a family, consisting of related people who may or may not live together. The compositional variables are based on only alters’ characteristics reported by household questionnaire respondents. These variables are indicators of a woman’s network of diverse alters and associated social capital resources [22].

The compositional variables were determined by the index of qualitative variation (IQV) on a continuous scale. IQV is defined as “the probability that a randomly selected pair of observations will be in different categories except that it’s maximum possible value is 1.0” (Dickinson and Gentry 1999). The IQV has an advantage over other diversity measures, e.g., the Blau’s index, as the latter cannot be compared across variables when variables have different categories. The IQV controls the number of categories in each variable, enabling the household diversity to be compared across variables [23].

The IQV value ranges from 0 to 1 (can be described as a percentage of 0 to 100%), with higher scores indicating more heterogeneity. When all alters are in the same category (e.g., if all alters are female or male), there is no diversity, and the score is zero. The even distribution of alters across categories maximizes diversity (e.g., if alters have equal males and females); the IQV is 1. This study has six compositional variables, including IQV of sex, IQV of educational status, and IQV of household members kinship, all treated as continuous variables. The age diversity of alters was estimated by using standard deviation. IQV of de jure members (usual household residents irrespective of their presence during the survey) and de facto family members (actual household members present in the household at the time of the survey irrespective of their usual residence or visitors status) were split based on the median values and coded as diversity present/absent. The summary of composition variables and each variable’s definition and analysis plan are in Supplementary File S2.

#### 2.4.3. Household Structures

The DHS records the relationship between each household member and the household head, used to estimate the ties among alters. The structural variables were calculated by considering the presence or absence of ties among alters, using their kinship status as a guide. The kinship status was estimated based on the coefficient of relatedness, scoring 0.5 for parents and children, 0.25 for grandparent and grandchild, 0.125 for nephew or nieces, and zero for non-related family members (dyadic by its nature) [24]. This means that the woman respondent has ties to her parents, but not her parents-in-law. Her husband will have ties to his parents, but not his parents-in-law. The child will be related by blood to both parents and both sets of grandparents and have ties to all of them.

The alter–alter ties were created by assigning the coefficient above of relatedness to alters. SPSS version 25 [25] produced a multiplicative matrix for the unique possible combination (a half matrix) among alters, assuming alter ties undirected. The detailed procedure is presented in Supplementary Materials File S3. This strategy can simultaneously ensure the tie’s presence or absence and weigh the tie when related.

Six structural variables, namely degree, density, effect size, efficiency, constraint, and hierarchy, were measured on a continuous scale, and measures are limited to alters. These variables were constructed from the presence or absence of blood relationships among alters. Household members connected to the women might not necessarily have blood relations, resulting in a missing link (structural hole). Such a missing link means that these people are aware of each other but differ in their level of trust and interaction, and they might not be obliged to support the women, but the missing link facilitates access to different information flows [26].

The above structural variables are overlapping; hence, a principal component analysis was run to obtain a representative but smaller set of variables with a degree, effective size, and constraint for inclusion in the final analysis (Supplementary File S3). The degree is the number of household members, including children and adults (alters), living with women. Constraints describe the extent to which alters are related to each other. The constraint score ranges from 0 to 1 (0 to 100 percentage points). When all alters are unrelated (no tie among alters), the score is 0. When all alters are nuclear family members, the constraint is 1. 

The effective size (ES) describes the non-redundancy of kinship types, and it measures the benefit received for every unit invested over alters. Effect size ranges from 1 (when all alters are related) to n (equal to the number of alters when all alters are unrelated). The nuclear family members have high redundancy and lead to a low ES, while non-relatives and extended families in the network increase the ES. The ES is dichotomized as high (>3) and low (≤3) effective size based on the median value. The details of operational definition, measurement level, and analysis plan of structural variables are provided in Table 1 and Supplementary File S3.

### 2.5. Primary Outcomes

The study’s primary outcomes were Minimum Dietary Diversity (MDD) and Minimum Meal Frequency (MMF). Survey-round-specific and pooled analyses were conducted.

#### 2.5.1. Minimum Dietary Diversity (MDD)

The infant- and young-child-feeding questionnaire asked about food consumed during the previous day across eight food groups, including breast milk; grains, roots, and tubers; legumes and nuts; dairy products and eggs; vitamin-A-rich fruits; and other fruits and vegetables. The dietary questionnaire asked only about food types, not amounts. The MDD indicator is constructed by summing all food groups consumed, with scores ranging from 0 (none consumed) to 8 (all consumed), and dichotomized into not meeting (0–4 food groups) versus meeting (5–8 food groups) the MDD recommendation with the updated WHO criteria [27,28]. The MDD has been validated and correlated with dietary adequacy for infants and young children [29].

#### 2.5.2. Minimum Meal Frequency (MMF)

MMF is the minimum number of times the child received solid, semi-solid, or soft foods (but also includes milk for non-breastfed children) over the previous day. MMF was analyzed as a dichotomous variable (meeting versus not meeting MMF recommendation). For breastfed children, meeting MMF is defined as twice a day for 6-to-8-month-olds and three times per day for 9–23-month-olds. For non-breastfed children 6–23 months, meeting MMF is defined as at least four times per day and that at least one of the feedings is solid, semi-solid, or soft foods, based on the updated guideline [27,28].

### 2.6. Statistical Methodology

The study has two sets of exposure variables: household composition (Supplementary File S2) and structural variables (Supplementary File S3). Other household characteristics (wealth index, sex of household head, and coresidence with husband), and the women’s demographic characteristics (age in year, educational status, types of earning, residence, and respondent relation to the child (maternal/non-maternal)) were considered as covariates (Supplementary File S1). The details of the variable’s measurement scale and analysis plan are provided in Supplementary Files S1–S3.

Statistical analysis was performed by using Stata version 13, with statistical significance set at *p* < 0.05. All analyses were adjusted for sampling weight to ensure that the results conform to the national estimates [30]. Descriptive analyses were conducted to summarize women’s characteristics and household composition and structures for each survey round. Normally distributed continuous variables were described with weighted mean, standard error, while non-normally distributed variables were described with median and 25th and 75th percentile.

Each structural and compositional variable was analyzed in separate models, as some of the variables were correlated. Univariate logistic regression was initially performed to evaluate the association between structural and compositional variables and complementary-feeding-practices outcomes (meeting versus not meeting the MDD and MMF recommendation). Additional logistic regression analyses adjusting for the aforementioned covariates were run for each structure and composition variable across survey rounds.

Multilevel mixed-effects logistic regression specifying survey year as a random effect to account for differences by survey round was used to obtain the combined effect size of the four survey rounds to identify the variable’s importance to inform policy and practice. The model was adjusted for the covariates above to obtain an overall adjusted odds ratio between each structural and compositional variable and each complementary feeding practice outcome for all survey rounds.

Based on the multilevel mixed-effects regression model, the average Marginal Effects (MEs) for each structural and compositional variable were calculated to facilitate clinical interpretation. The ME was interpreted as a predictive probability of change (multiplied by 100 and reported as a percentage change points) in the outcome variable associated with one standard-deviation increase above the mean value for continuous explanatory variables or incremental change from the reference value for categorical variables [31].

## 3. Results

### 3.1. Participant Characteristics 

**Figure 2:**
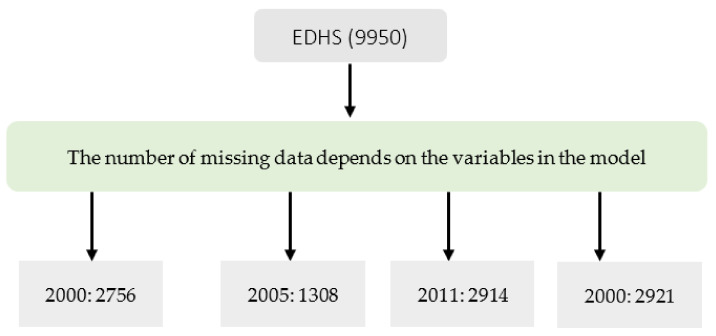
Number observations analyzed (unweighted observation) from Ethiopian Demographic and Health Survey (EDHS).

This secondary-data analysis was based on 9950 observed cases (weighted cases: 10,750) of last-born children between 6 and 24 months. The number of analyzed cases varies for each outcome. MDD results were available for EDHS 2000, 2005, and 2016; MMF results were presented for EDHS 2005, 2011, and 2016 (Figure 2).

The survey respondents had a comparable age across survey rounds, with a mean age of 28 years. Women with primary-school education and above ranged from 12% to 38%. The women-headed households increased from 10% in 2000 to 16% in 2016, with only a third of women in paid work. Respondents were predominantly maternal (86.2% to 90%). The proportion of households in the poor wealth tercile ranged from 17.7% to 38% (Table 2).

The median number of people (degree) living with women was five, with the interquartile range (IQR) of three, except for EDHS 2005. The median effective size (the number of women contacts adjusted for related household members) was three (expected to be five, the degree if all alters were unrelated). The women’s median constraints were 0.56 across the survey (i.e., 56%; if all contacts were related, the score would be 100%, but the network comprises 56% of ties of related alters, showing the presence of unrelated/extended family members). The percentage of sex differences in male and female alters was 0.89 (i.e., 89%) of the maximum possible gender differences of 100% if there was an equal split; the IQV of household members’ kinship ranges from 0.75% to 0.89%, implying that the women contacts were diverse in their relationships (Table 2).

The percentage of children meeting the MDD was lowest in 2011, at 4.2%, and highest in 2016, at 12.4%. The percentage of children meeting MMF was lowest in 2000, at 43.5%, and highest in 2011, at 50.2%.

### 3.2. Factors Associated with Complementary Feeding Practices

#### 3.2.1. Minimum Dietary Diversity (MDD)

Adjusting for the women characteristics and the wealth index, we found that the degree (the number of alters) seemed to increase overall MDD (Table 3) (Marginal Effect (ME) = 0.11%; 95% CI: −0.62%, 0.83%). Moreover, there was no difference in probability of meeting the MDD (ME = 1.01%; 95% CI: 0.81%, 1.25%) between those with high (≥three kinship categories) and low effect size (<three kinship categories). Household constraint (the more closely related alters) appeared to increase the proportion of children meeting the MDD by 1.20% (95% CI: −1.09%, 0.17%).

Regarding the diversity of household composition, the proportion of children meeting the MDD increased by 0.13% (ME = 0.13%, 95% CI: 0.10%, 0.17%) as the age diversity increases; that is, the more mixed-age alters there are, the higher the probability of meeting the MDD. The IQV of educational status tended to increase the probability of meeting the MDD (ME = 1.39%; 95% CI: −1.22%, 3.79%), showing that the presence of alters with various educational status increases the overall probability of meeting the MDD.

Concerning the IQV of de jure household members, compared to a household with no diversity, the proportion of children meeting the MDD in households with diversity (the presence of at least one visitor) was 1.23% higher (95% CI: −1.25%, 3.72%), although not statistically significant. This implies that the presence of visitors positively influences the MDD (Table 3). Similarly, going from low to high IQV of de facto family members (presence of usual household residents who have not slept last night at the households) seemed to increase the overall probability of meeting the MDD (Table 3). The IQV of kinship types, a caregiver with various kinships category, seemed to decrease the proportion of children meeting the MDD by 1.31% (95% CI: −7.22%, 4.60%).

#### 3.2.2. Minimum Meal Frequency (MMF)

Adjusting for the women characteristics and the wealth index, the degree (the number of alters) significantly reduced the proportion of children meeting MMF by 0.82% (95% CI: −1.22%, −0.42%). However, relative to low effect size, households with high effective size significantly reduced the proportion of children meeting MMF by 3.19% (95% CI: −5.12%, −1.26%). The significant effect was also observed for constraint, where a unit change increased the probability of meeting MMF (ME = 13.70%, 95% CI: 8.43%, 18.97%), suggesting that households made of related people, either by birth or marriage, were more likely to meet MMF compared to those made of unrelated people. Regarding compositional variables, IQV of de facto household members, usual members away from home, increased the proportion of children meeting MMF by 3.61% (95% CI: 0.16%, 7.06%). On the other hand, IQV of de jure household members, the presence of visitors, decreased the proportion of children meeting MMF by 3.62% (95% CI: −7.15%, −0.09%) (Table 4).

### 3.3. Variation by Survey Round

The association between the structure and composition variables and MDD shifted from positive to negative or vice versa in the first two rounds of EDHS to a positive in EDHS 2016, except for IQV of kinship types. There was a consistently positive association between the MMF and constraints but a negative relationship with effect size and degree. There were variations in the association between MMF and the alter composition variables, which turned positive in EDHS 2016, except for age diversity.

## 4. Discussion

This study examined how Ethiopian household structure and composition and accompanying changes between 2000 and 2016 influenced complementary feeding practices, measured as MDD and MMF. Overall, the number alters, including the number of unique kinship categories, positively influenced the MDD and negatively the MMF except for households made of close kins. Household diversity in terms of age, sex, educational status increased MDD and decreased MMF. Both MMD and MMF were decreased with more kinship diversity and increased with members staying away from home.

While evidence suggests there has been a reduction in suboptimal complementary feeding practices in Ethiopia, the problem remains unacceptably high, with less than one in ten children meeting both MDD and MMF, threatening the Seqota Declaration, Ethiopia’s commitment to ending child stunting by 2030 [32]. The declaration’s priority interventions are directed to the household; however, it was not explicitly stated whom to involve beyond the traditional child caregivers in the household [32].

Similarly, the Ethiopian food and nutrition policy recognizes the need to enhance household knowledge in childcare to optimize feeding practices. However, it does not specify the actions, contact points, and strategies to reach each household member (Federal Democratic Republic of Ethiopia). Previous EDHS analyses of child-feeding practices focused on maternal, child, or paternal characteristics [5,33] and did not take a holistic view of the women’s support system within the household, influenced by the composition and structure of the household. This is the first study to explore these dimensions by using large scale national data.

Overall, the number of people living in the respondent’s households has not changed between 2000 and 2016. Moreover, the effective size, the number of people with unique relationships living with the women, remained unchanged at three people. The difference between degree and effective size shows redundant relationships among women’s contact, which was accounted for in the effect size, indicating that the women’s supports are potentially more additive than overlapping. The non-redundancy might arise from the composition of typical rural Ethiopian households of three generations: the eldest couple, their sons, sons’ wives, unmarried daughters, and grandchildren from their married sons. However, urban households have a nuclear family’s structure consisting of parents and their children [34].

The household network under investigation tended to have high constraint scores, indicating that the household was made up of relatives [35]. The consequence of high constraint is that the women potentially invest substantial time and effort in the children and other household members. In context, Ethiopian women are primarily responsible for domestic activities imposed by gender relations [36], which might have multiple positive and negative child-feeding implications, as discussed below.

The constraint positively influenced MDD and MMF, but was most substantial for MMF. This is consistent with Hamilton’s kin selection theory, which states that families have better collaboration, cooperation, and altruism, leading to positive outcomes, including for children [37,38,39]. The high level of constraint might accompany bonding social capital, associated social support that translated to better complementary feeding practices. This might indicate the potential of a family-centerd approach aimed at nuclear households, the parents, and siblings.

The mechanism by which constraints help improve complementary feeding can explain how family members support the women. For instance, in Ethiopia, grandmothers customarily visit their daughters and are involved primarily in providing childcare than help with domestic work [40]. The prioritizing of childcare may cause more competition with childcare but add experiences to feeding practices. Similarly, the Western Kenya experience shows that fathers and grandmothers help improve the knowledge of optimal infant-feeding practices [41].

The overall constraint had no or negative effect on MDD in earlier survey rounds but shifted to positive for EDHS 2016. The shift can be viewed from the structural hole perspective, which argues that fewer holes (households composed of more related people) are associated with fewer advantages, because it limits people’s access to diversified information sources [35,42]. Hence, it can be stated that, for earlier EDHS rounds, structural holes are the mechanism of social capital that negatively influences MDD. Thus, weak ties of unrelated individuals improve MDD by facilitating access to external resources, such as sharing knowledge and bridging social capital [43].

The positive relationship between MDD and constraints in 2016 and MMF and constraints across the survey might be explained with closure, a mechanism behind social capital where households with closely related people have more reinforcement to get support [42]. In turn, that facilitates direct access to helpful information. Compared to previous surveys, during EDHS 2016, households may have had better access to information that changed or shaped norms surrounding complementary feeding practices. The change might be consistent with the government’s strategies: active community participation and mass-media campaigns [44,45,46].

Thus, the complementary feeding information is to be targeted to all household members. The ‘where’ aspect of the intervention might range from home to schools and other institutions, customized for delivering feeding and caregiving resources, including education to bring changes in feeding and caregiving practices, parenting, and reinforcement [47]. Specifically, in the intermediate-to-long-term, the Government of Ethiopia can design a curriculum to deliver gender-responsive age-appropriate education on child feeding, grounding the understanding in the future generation. Moreover, other nutrition interventions must engage beyond usual household members who play a crucial role [48].

On the other hand, MMF was negatively associated with the degree and effective size but positively associated with constraint. The more people in the household, the less likely children were to meet MMF, but this was attenuated if those household members were closely related, as in the nuclear family type. This finding might reflect household food security [47]. Intervention that helps to target these underlying causes, such as agriculture and food security, social safety nets, and health and family planning services, might benefit from considering the household structure.

The household diversity in terms of actual and usual residents denotes people’s movement away and to the household, respectively. Our finding showed a positive association between members staying away from home and MDD and MMF, which might be related to less family to feed and more people earning. The positive association is consistent with the empirical evidence from Ethiopia, where rural outmigration significantly reduced household food insecurity [49]. On the other hand, visitors at the household were positively associated with MMD; customarily, a guest’s arrival might positively alter the diversity of the diet with a range of food offered but with a negative effect on the MMF [49].

The existing Ethiopian nutrition intervention approach is multi-sectoral coordination, including agriculture, health, education, and other sectors [48]. At the grassroots level, the delivery of a household-centered approach might be reinforced by tailor-made communication materials that account for sociodemographic diversities, such as sex, age, educational status, and usual and actual household residents. For instance, contextual factors to consider might include people who cannot read, traditional male-dominated households, extended family households that prioritize the older family members’ views, young or first-time parents, caregivers on behalf of parents, and those who are around the child frequently or are away. Thus, future interventions might benefit from expanding from a family-centered to household-centered approach to align with the positive influence of visitors and usual residents away from home [41].

The development of contextualized materials needs further investigation, yet it can be implemented by the Health Extension Workers and the Health Development Army (HDA) leaders; the key actors are the Ethiopia social network platforms and the HDA. The educational sector is a multi-sectoral platform for mainstreaming nutrition in the school curriculum [48]. However, the level of early nutrition mainstreaming in the curriculum needs further investigation. Moreover, given the magnitude of the problem, a separate course, such as home economics, can tackle priority nutrition problems, including complementary feeding practices.

Finally, this study has strengths and weaknesses. The strengths include using the social network analysis with large scale data; using complex survey design facilitates the representativeness of the findings to the larger population and comparisons of outcomes across survey years. However, in most cases, the associations are in the expected direction but not statistically significant, due to complex survey design that increases the standard errors of estimates and a small proportion in some of the categories of variables [50].

## 5. Conclusions

In conclusion, to promote optimal complementary feeding practices, interventions should be adapted to account for the variations in the household structure and composition. That is, interventions might be repositioned to address each household member, beyond families, to empower him or her to make the right feeding decisions when delegated to assume caregiving roles, deliver better support to primary caregivers, and use the skill for his/herself. Future studies might compare how well complementary feeding interventions work across households with variable compositions and structures.

## Figures and Tables

**Table 1:** Summary of scoring of structural variables.

Variable	Operational Definition	Score
Degree	The number of household members the woman respondent lives with.	Ranges between 1 and n, where n is household size, excluding the women.
Effective size	The non-redundant alters in the women network, or it is a degree adjusted for redundancy of kinship types.	Ranges between 1 (all alters related) and n (when all alters are unrelated), where n is household size, excluding the women.
Constraints	It describes how the women are connected to related alters (family members), controlling for non-redundancy of kinship types among alters.	Ranges from 0 (where all alters are unrelated) to 1 (where all alters are related).

**Table 2:** Participant characteristics of households with children 6–24 months, EDHS 2005 to 2016 (weighted).

Variables	Categories	2000 (*n* = 3250)	EDHS 2005 (*n* = 1423)	2011 (*n* = 3022)	2016 (*n* = 3055)
		*n* (%) ^d^	*n* (%)	*n* (%)	*n* (%)
Women characteristics					
Women age in years (mean ±SE)		28.3 (0.21)	27.7 (0.26)	27.9 (0.18)	27.8 (0.20)
Educational status	No formal education	2859 (88)	878 (61.69)	2173 (71.9)	2102 (68.8)
	Primary and above	390 (12)	546 (38.31)	849 (28.1)	952 (31.2)
Residence	Urban	216 (6.6)	536 (37.6)	476 (15.8)	593 (19.4)
	Rural	3034 (93.4)	888 (62.4)	2546 (84.2)	2462 (80.6)
Sex of household head	Female	320 (9.9)	227 (15.9)	506 (16.7)	484 (15.9)
	Male	2930 (90.1)	1197 (84.1)	2516 (83.3)	2570 (84.1)
Type of earnings	Not working	983 (30.3)	827 (58.1)	1989 (65.8)	1719 (56.3)
	Working but not paid	973 (29.9)	119 (8.3)	317 (10.5)	397 (13.0)
	Paid in cash in-kind	1292 (39.8)	478 (33.6)	716 (23.7)	937 (30.7)
Household structure					
Degree (median; Q1, Q3)		5 (3, 6)	4 (3, 6)	5(3, 6)	5 (3, 6)
Effective size (median; Q1, Q3)		3 (2.17, 4)	3 (2,4)	3 (2.33, 4)	3 (2.5,4)
Constraints (median; Q1, Q3)		0.56(0.44, 0.65)	0.55 (0.44, 0.71)	0.56 (0.44, 0.64)	0.55 (0.44, 0.64)
Wealth index	Poor	1237 (38.1)	252 (17.7)	1036 (34.3)	1015 (33.2)
	Middle	1210 (37.2)	349 (24.5)	968 (32.0)	1048 (34.3)
	Rich	803(24.7)	823 (57.8)	1018 (33.7)	992 (32.5)
Household composition					
Co-residence with husband	Living with women	2671 (91)	2431 (93.1)	2622 (88.7)	1166 (88.3)
	Stay elsewhere	264 (9)	179 (6.9)	335(11.3)	155 (11.7)
Types of the respondents	Non-maternal caregivers	332 (10)	196 (13.8)	313 (10.3)	382 (12.5)
	Maternal caregivers	2917 (90)	1228 (86.2)	2709 (89.7)	2672 (87.5)
SD of age in year (median; Q1, Q3)		13.68 (11.9, 16.9)	13.5 (11.32, 16.47)	13.16 (11.14, 16.03)	13.02 (11.15, 16.31)
IQV^€^ of sex (median; Q1, Q3)		0.89 (0.69, 0.97)	0.89 (0.64, 0.98)	0.89 (0.75,0.98)	0.89 (0.75, 0.98)
IQV of educational status (median; Q1, Q3)		0 (0, 0.75)	0.75 (0,0.94)	0.75 (0,0.96)	0.82 (0,0.96)
IQV of de facto household members	Diversity absent	2735 (84.2)	1215 (85.5)	2594 (85.9)	2602 (85.2)
	Diversity present	515 (15.8)	206 (14.5)	426 (14.1)	452 (14.8)
IQV of de jure household members	Diversity absent (ref)	2813 (86.6)	1276 (89.82)	2801 (92.74)	2731 (89.4)
	Diversity present	437 (13.4)	145 (10.18)	219 (7.26)	3234 (10.6)
IQV of kinship types		0.75 (0.64, 0.94)	0.89 (0.64,0.94)	0.75 (0.56, 0.93)	0.75 (0.56, 0.92)
**Complementary feeding outcomes** *					
Minimum Dietary Diversity	No	-	1246 (94.86)	2709 (95.76)	2502 (87.60)
	Yes	-	67 (5.14)	120 (4.24)	354 (12.4)
Minimum Meal Frequency	No	1709 (56.5)	-	1408 (49.8)	1459 (51.1)
	Yes	1315 (43.5)	-	1420 (50.2)	1395 (48.9)

* Items needed to construct MDD and MMF were not collected in 2000 and 2005, respectively. Hence, their composite variable, MAD, is calculated for EDHS 2011 and 2016. ^d^ The values are presented as n (%) unless specified. Abbreviations: EDHS, Ethiopian Demographic and Health Survey; MDD, Minimum Dietary Diversity; MMF, Minimum Meal Frequency; SE, standard error; Q, quartile; IQV, index of qualitative variation.

**Table 3:** Factors associated with an MDD, EDHS 2005 to 2016 ^∋^.

Variables	Categories	EDHS Round						Overall AOR ^g^	Marginal Effect (%) ^y^
		2005		2011		2016			
		COR ^€^ (CI)	AOR ^d^ (CI)	COR (CI)	AOR (CI)	COR (CI)	AOR (CI)		
**Household structure**									
Degree		0.92 (0.82, 1.04)	0.96 (0.84, 1.09)	0.93 (0.82, 1.06)	0.88 (0.73, 1.06)	1.04 (0.96, 1.12)	1.06 (0.97, 1.16)	1.02 (0.92, 1.12)	0.11 (−0.62, 0.83)
Effective size ^δ^	Low effect size (reference)								
	High effect size	0.87 (0.43, 1.77)	1.06 (0.49, 2.32)	0.81 (0.46, 1.42)	0.72 (0.39, 1.35)	1.08 (0.76, 1.53)	1.11 (0.78, 1.57)	1.01 (0.81, 1.25)	0.04 (−1.41, 1.48)
Constraints		1.93 (0.50, 7.54)	1.00 (0.17, 5.92)	1.00 (0.17, 5.83)	0.93 (0.1, 8.33)	1.26 (0.39, 4.02)	1.32 (0.36, 4.87)	1.20 (0.95, 1.51)	1.20 (−1.09, 3.49)
**Household composition**									
Age diversity (SD of age in years year)		1.01 (0.94, 1.10)	1.03 (0.96, 1.11)	1.03 (0.99, 1.08)	1.02 (0.97, 1.08)	1.01 (0.97, 1.06)	1.01 (0.97, 1.06)	1.02 *** (1.01, 1.03)	0.13 *** (0.10, 0.17)
IQV of sex		1.03 (0.43, 2.48)	1.22 (0.48, 3.11)	1.03 (0.46, 2.31)	0.99 (0.44, 2.23)	1.31 (0.76, 2.26)	1.34 (0.82, 2.2)	1.20 (0.98, 1.47)	1.20 (−0.87, 3.26)
IQV of educational status		0.53 (0.24, 1.14)	0.74 (0.29, 1.93)	0.97 (0.51, 1.84)	1.02 (0.55, 1.92)	1.41 (0.85, 2.34)	1.55 (0.90, 2.69)	1.23 (0.82, 1.85)	1.39 (−2.13, 4.91)
IQV of De facto household members	Diversity absent (reference)								
	Diversity present	0.44 (0.13, 1.50)	0.48 (0.13, 1.74)	1.96 * (1.02, 3.78)	1.92 (0.98, 3.76)	1.15 (0.70, 1.89)	1.17 (0.71, 1.91)	1.20 (0.83, 1.73)	1.28 (−1.22, 3.79)
IQV of De jure household members	Diversity absent (reference)								
	Diversity present	1.96 (0.86, 4.44)	2.49 * (1.07, 5.8)	1.20 (0.47, 3.03)	1.17 (0.4, 3.4)	0.97 (0.51, 1.82)	1.00 (0.52, 1.91)	1.19 (0.79, 1.79)	1.23 (−1.25, 3.72)
IQV of kinship types		0.78 (0.18, 3.42)	0.77 (0.14, 4.33)	2.93 (0.81, 10.61)	2.78 (0.29, 26.68)	0.73 (0.37, 1.46)	0.58 (0.26, 1.3)	0.82 (0.38, 1.78)	−1.31 (−7.22, 4.60)

COR, crude odds ratio; AOR, adjusted odds ratio; * *p* < 0.05; ** *p* < 0.01; *** *p* < 0.001. ^∋^ The items needed to construct MDD were not collected during EDHS 2000. ^€^ Crude model includes each family structure and composition variable as the exposure and MDD as the outcome, using -svy-. ^d^ Adjusted model adjusted for covariates, including women age in years, educational status, residence, coresidence with husband/partner, sex of household head, wealth index, and type of earnings, using -svy-. ^g^ Overall OR was calculated with multilevel mixed-effects logistic regression modeling, specifying survey year as a random effect. ^y^ The average Marginal Effect was calculated for each variable as the discrete change from the reference value for categorical variables (effective size, de facto family members, and de jure family members) and a probability of meeting MDD with a small change for continuous variables from the multilevel mixed-effects logistic regression model. ^δ^ Low effect size ≤ 3 kinship categories; high effective size < 3 kinship categories.

**Table 4:** Factors associated with an MMF, EDHS 2000 to 2016 ^∋^.

Variables	Categories	EDHS Round						Overall AOR ^g^	Marginal Effect (%) ^y^
		2000		2011		2016			
		COR ^€^ (CI)	AOR ^d^ (CI)	COR (CI)	AOR (CI)	COR (CI)	AOR (CI)		
**Household structure**									
Degree		0.96 (0.91, 1.01)	0.97 (0.92, 1.02)	1.00 (0.96, 1.05)	0.98 (0.92, 1.04)	1.01 (0.96, 1.05)	0.96 (0.9, 1.01)	0.97 *** (0.95, 0.98)	−0.82 *** (−1.22, −0.42)
Effective size ^δ^	Low effect size (reference)								
	High effect size	0.85 (0.69, 1.05)	0.90 (0.73, 1.12)	0.98 (0.78, 1.23)	0.9 (0.7, 1.18)	0.99 (0.81,1.20)	0.81 (0.64, 1.04)	0.88 ** (0.81, 0.95)	−3.19 ** (−5.12, −1.26)
Constraints		1.93 * (1.04, 3.58)	1.55 (0.7, 3.48)	1.10 (0.57, 2.13)	1.63 (0.83, 3.22)	0.93 (0.67, 1.31)	2.07 * (1.03, 4.18)	1.74 *** (1.41, 2.16)	13.70 *** (8.43, 18.97)
**Household composition**									
Age diversity (SD of age in years year)		0.99 (0.97, 1.01)	0.99 (0.97, 1.02)	1.00 (0.98, 1.02)	1.00 (0.98, 1.03)	1.00 (0.98, 1.03)	0.99 (0.97, 1.02)	1.00 (0.99, 1.00)	−0.10 (−0.24, 0.04)
IQV of sex		0.74 (0.53, 1.04)	0.79 (0.56, 1.12)	0.81 (0.58, 1.12)	0.78 (0.56, 1.1)	1.25 (0.89, 1.75)	1.09 (0.76, 1.57)	0.87 (0.70, 1.08)	−3.50 (−8.87, 1.87)
IQV of educational status		0.71 * (0.55, 0.92)	0.76 * (0.58, 0.99)	0.87 (0.65, 1.17)	0.84 (0.62, 1.14)	1.24 (0.93, 1.65)	1.31 (0.94, 1.81)	0.95 (0.70, 1.29)	−1.19 (−8.67, 6.29)
IQV of de facto household members	Diversity absent (reference)								
	Diversity present	1.00 (0.77, 1.32)	1.03 (0.79, 1.35)	1.26 (0.93, 1.69)	1.25 (0.92, 1.7)	1.27 (0.93, 1.74)	1.28 (0.93, 1.77)	1.16 * (1.01, 1.33)	3.61 * (0.16, 7.06)
IQV of de jure household members	Diversity absent (reference)								
	Diversity present	0.77 (0.56, 1.06)	0.85 (0.62, 1.17)	0.74 (0.49, 1.13)	0.77 (0.51, 1.18)	0.99 (0.69, 1.41)	1.00 (0.69, 1.45)	0.86 * (0.75, 1.00)	−3.62 * (−7.15, −0.09)
IQV of kinship types		0.94 (0.58, 1.52)	0.56 (0.3, 1.07)	0.71 (0.45, 1.11)	1.07 (0.58, 1.97)	0.99 (0.63, 1.57)	1.36 (0.7, 2.65)	0.99 (0.61, 1.58)	−0.36 (−12.06, 11.33)

COR, crude odds ratio; AOR, adjusted odds ratio; * *p* < 0.05; ** *p* < 0.01; *** *p* < 0.001. ^∋^ The items needed to construct MMF were not collected during EDHS 2005. ^€^ Crude model includes each family structure and composition variable as the exposure and MDD as the outcome, using -svy-. ^d^ Adjusted model adjusted for covariates, including women age in years, educational status, residence, coresidence with husband/partner, sex of household head, wealth index, and type of earnings, using -svy-. ^g^ Overall OR was calculated with multilevel mixed-effects logistic regression modeling, specifying survey year as a random effect. ^y^ The average Marginal Effect was calculated for each variable as the discrete change from the reference value for categorical variables (effective size, de facto family members, and de jure family members) and a probability of meeting MMF with a small change for continuous variables from the multilevel mixed-effects logistic regression model. ^δ^ Low effect size ≤ 3 kinship categories; high effective size < 3 kinship categories.

## Data Availability

Data are available upon request due to the Demographic and Health Survey Program restrictions. The data are available at https://www.dhsprogram.com/data/dataset_admin/login_main.cfm (accessed on 3 December 2020). Data citation: Elizabeth Heger Boyle, Miriam King, and Matthew Sobek. IPUMS Demographic and Health Surveys. Version 8 [dataset]. Minneapolis, MN: IPUMS and ICF.2020. https://doi.org/10.18128/D080.V8 (accessed on 15 December 2020).

## Supplementary Materials

The following are available online at https://www.mdpi.com/article/10.3390/nu14010130/s1, Supplementary File S1: The respondent and household characteristics measurement scale and analysis plan. Supplementary File S2: Alter composition variables and their operational definition. Supplementary File S3: Procedures to create the alter-alter ties and operational definitions of resulting structural variables.
