# Supplementary material for: The Role of Household Structure and Composition in Influencing Complementary Feeding Practices in Ethiopia
Source: Nutrients. 2021 Dec 28;14(1):130. doi: 10.3390/nu14010130 (PMC8747051; doi:10.3390/nu14010130)
Supplement: Supplementary file 1 [file nutrients-14-00130-s001.zip › nutrients-1511747-supple.pdf]

## Supplementary files

**Supplementary File S1.** The respondent and household characteristics measurement scale and analysis plan

| Variables | Measurement scale | Analysis plan |
|---|---|---|
| Women age | Measures in a continuous scale in the year(s) | It is approximately normally distributed, and it was described with mean and SE along with a 95% CI. |
| Women educational status | Measured on an ordinal scale:<br><br>0=no education<br>1=primary education<br>2=secondary education<br>3=higher education | The small categories (primary, secondary and higher education was merged as primary education and above). The recoded category is:<br><br>0=no education<br>1=primary education and above education<br><br>The variables will be described with number, percent, along with a 95% confidence interval. |
| sex of head of household | Measured on a nominal scale:<br>0=female<br>1=male | The variable was described with number, percent, along with a 95% confidence interval. |
| Types of earning | The original variable was measured on a categorical scale as follow:<br>0=Not paid<br>1=Paid in cash only<br>2=Paid in cash and in-kind<br>3=Paid in kind only<br>4=NIU (not in the universe): not working | The small categories (1=Paid in cash only, paid in cash and in-kind, paid in kind was merged). The recoded category is:<br><br>0=not paid<br>1=paid in cash or in-kind or both<br>2=not working<br><br>The variable was described with number, percent, along with a 95% confidence interval. |
| Residence | The residence is measured on a nominal scale:<br>1=urban<br>2=rural | The variable was be described with number, percent, along with a 95% confidence interval. |
| Women's relation to the head of household | The varible is measured in nominal scale as follow:<br>1=Head<br>2=Wife<br>3=Daughter or son<br>4=Daughter- or son-in-law<br>5=Grandchild<br>6=Mother or father<br>7=Parent-in-law<br>8=Sister or brother<br>9=Adopted/foster/step child | The variable category head and 'wife' was coded as maternal caregivers and the rest non-maternal caregivers as follow:<br>1=Maternal caregivers<br>2=Non-maternal caregivers<br><br>The variable was described with number, percent, along with a 95% confidence interval. |

| | | |
|---|---|---|
| | 10=Adopted/foster child<br>11=Other relative<br>12=Niece or nephew unspecified<br>13=Nonrelative | |
| **Family members-child dyads** | | |
| Coresidence with husband/partner | This variable is measured on the nominal scale as follow:<br><br>1=Living with women (ref)<br>2=Stay elsewhere | The variable was described with number, percent, along with a 95% confidence interval. |

**Supplementary File S2:** Alter composition variables and their operational definition

| **Household composition (ignoring the respondent)** | **Operational def. /rationale** | **Analysis plan** |
|---|---|---|
| IQV of household members relationship | Represent the presence of multiple generation household members and non-relatives. Measured on a continuous scale ranging from 0 to 1; the higher the score, the more diverse the alters.<br><br>The index show diversity in terms of the presence of people with multiple relationship statuses (head, wife, daughter or son, daughter- or son-in-law, grandchild, mother or father, parent-in-law, sister or brother, adopted/foster/stepchild, adopted/foster child, another relative, niece or nephew unspecified, non-relative). | The variable has non-normal distribution; thus, described with the median, 25th and 75th percentile and will be added in the model as a continuous variable. |
| IQV sex | Family members sex diversity is measured on a continuous scale, ranging from 0 to 1; the higher the score, the higher sex diversity, meaning alters are classified fairly across the male and female categories. | The variable has non-normal distribution; thus, described with the median, 25th and 75th percentile and will be added in the model as a continuous variable. |
| IQV de jure alters | The measure is based on how the household is diversified on whether alters are usual residents or not.<br><br>Usual residence in the household might attach to specific roles and responsibilities, including child feeding, depending on members availability, age, sex, and other household members attributes. When people are away, the roles must be delegated to someone else (Bender, 1971). | The variable has a non-normal distribution with the median value, 25th and 75th percentile of zero.<br><br>Hence, the variable is dichotomised based on the median (median split)—score £0 coded zero, everyone is usual resident, and the value >0 coded 1 indicates people who are not usually resident. The variables are labelled as follow:<br><br>0= diversity absent (all alters are usual residents)<br>1= diversity present (at least one alter a visitor )<br><br>The variables will be described with number, percent, along with a 95% confidence interval. |

| | | |
|---|---|---|
| IQV de facto alters | These people actually slept in the household the night before the data collection.<br>Despite people's usual residence at women households, they might not live with her, indicating weak ties irrespective of their kinship status, but the women might tap unique support from members who stay away from home.<br><br>Why usual/visitor status matter:<br>1. Sometimes, people eat in one household and sleep in another. The person is considered to be a member of the household where he/she sleeps.<br>2. A person living alone in a household with a child, where visitors matter the most<br>3. A woman may list her husband as head of the household, but he lives somewhere else. If he does not usually live in the household and did not sleep there the previous night, he will not be included in the listing. | The variable has a non-normal distribution with the median value, 25th and 75th percentile of zero.<br><br>Hence, the variable is dichotomised based on the median (median split)—score £0 coded zero, everyone stayed at household the previous night, and the value >0 coded 1 indicates people who have not spent the previous night. The variables are labelled as follow:<br><br>0= diversity absent (all listed alters slept in the household the night before the data collection)<br>1= diversity present (at least one usual resident slept in the household the night before the data collection)<br><br>The variables will be described with number, percent, along with a 95% confidence interval. |
| IQV Educational status | The educational status diversity index was constructed from no education, primary education, secondary education, or higher education of alters.<br><br>Education status diversity score is 1 when alters are equally distributed across the four categories and zero if alters have similar educational status. | The variable has non-normal distribution; thus, described with the median, 25th and 75th percentile and will be added to the model as a continuous variable |
| The standard deviation (SD)of age in years | Household members age diversity in years<br><br>When the SD of age increases, the age diversity increase. | This variable is approximately normally distributed; hence, it will be described with mean and standard error (SE) |

**Supplementary File S3:** Procedures to create the alter-alter ties and operational definitions of resulting structural variables.

**Procedures to create the alter-alter tie**

The DHS household roster used to collect the data contains 20 lines (family members), including the women respondents. That gives a 380 possible combination of [n(n-1), where n is the number of alters]. The ties are assumed undirected (the upper and lower half is a mirror image for each other); hence, the matrix gave 190 unique combinations [n(n-1)/2)], saved as a variable to calculate social network measures.

| ›usehold members | a1 | a2 | a3 | a4 | a5… | a20 |
|---|---|---|---|---|---|---|
| **a1** | | | | | | |
| **a2** | a1*a2 | . | | | | |
| **a3** | a1*a3 | a2*a3 | . | | | |
| **a4** | a1*a4 | a2*a4 | a3*a4 | . | | |
| **a5…** | a1*a5… | a2*a5… | a3*a5… | a4*a5… | . | |
| **a20** | a1*a20 | a2*a20 | a3*a20 | a4*a20 | a5*a20 | . |

In the same token, suppose a household with five family members, the mother (ego), father, two children (adopted and biological ) and a grandmother (women side). The following is the matrix obtained from the coefficient of relatedness, scoring 0.5 for parents and children; 0.25 for grandparent and grandchild; 0.125 for nephew or nieces, and zero for non-related family members (dyadic by its nature).

| Coefficient of relatedness | father | Adopted child | Biological child | grandmother |
|---|---|---|---|---|
| father | . | | | |
| Adopted child | 0 | . | | |
| child | 0.5 | 0 | . | |
| grandmother | 0 | 0 | 0.25 | . |

The egonet generates different network illustrations (Figure S1), and the following table explain the variables.

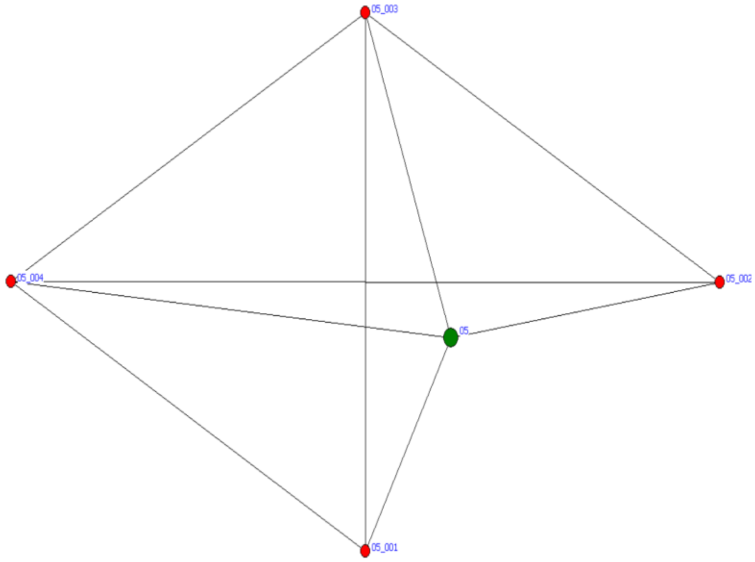


Figure.S1 Illustration for the intrahousehold network generated by egonet (green is the women respondent; red represent the alters)

| **Structural variables** | **Operational def. /rationale** |
|---|---|
| Degree (network size) | It describes the number of household members living with the woman. It differs from household size as it excludes the women respondent (ego). It denotes the network size. |
| Density<br><br>(Munoz-Carril et al., 2019) | It shows how connected the alters are compared to how related they might be.<br><br>For instance, in Figure S1, the number of alters is 5, then each alters can potentially connect to 4 other alters, but the actual connection depends on the kinship status, absent when a member is a non-relative.<br><br>Density ranges from 0 to 1, where 1 denotes everybody related to everyone.<br><br>The larger the value, alters are mainly relatives and potentially provide redundant information to the women; this could be viewed positively if the women need social support. However, it also imposes more control and sanction and is inefficient for creative new solutions such as improved feeding practices. High-density shows that alter related to each other.<br><br>Density can also tell how the women network structures vary across households. |
| Effective size<br><br>(Crossley et al., 2015) | This is an alternative way of measuring the network size (degree), but controls for redundancy of relationship among alters category showed in density. Effective size can be described as the number of alters the women are connected to minus duplicate ties among them.  Thus, since the redundant ties are accounted for, the effect size is much smaller than the actual network size.<br><br>The effective size can range from 1 (women with one alter, possibly only her child) to *N* (all alters are non-redundant). |

| | |
|---|---|
| | A woman with a larger effective size is associated with diversified ties, thus better social support.<br><br>For regression analysis, the effect size, which has a high correlation with degree and constraints, was dichotomised based on the median as low effect size ≤3 and high >3. |
| Efficiency<br><br>(Crossley et al., 2015) | The efficiency of an actor's network is computed as the effective size divided by the number of actors in the network, or it is the normalised version of effective size.<br><br>To put it in perspective, maintaining ties requires resources, and the women should wisely decide to whom to reach out for alternative views, which is naturally obtained from unconnected alters, the unrelated people/visitors. |
| Constraint<br><br>(Crossley et al., 2015) | It describes how the women are connected to related household members (e.g. members are limited to a child/son or in-laws) or a dense network. The positive or negative influence of constraints on complementary feeding practices would be interpreted based on the mechanisms that provide social support: the structural hole (absence of ties) and closure (closely knitted ties).<br><br>Structural holes: the women have a variable number of household members with different relations (including unrelated, meaning missing link among actors). This reduces constraints but increases the diversity of support available to the women.<br><br>Closure: On the other hand, if closure (closely knitted ties) are considered the source of support, more constraints might be associated with support.<br><br>For instance, if all alters have a close relation and live together, they are more likely to invest in their relation (time and energy), reducing the diversity of support the women get (constraints the women). On the other hand, if kinship is considered the primary support source, the same structure might provide more support. |
| Hierarchy<br><br>(Crossley et al., 2015, Burt, 2000) | It describes the nature of constraint imposed on the women, whether constraint comes from a single alter (higher hierarchy score) or constraints is distributed equally among alters (low hierarchy score).<br>This measures the level of dependency. The hierarchy score is zero when all alters disconnected from one another or connected.<br><br>For instance, if two or more household members are not-related (not-connected) but connected indirectly through a third household member:<br><br>Consider a case of the relationship between maternal and paternal side grandmothers connected through the child.When the child is a young caregiver, the child shares the same experience of child feeding on both sides as the primary caregiver (the ego).<br><br>Child feeding suggestions from both sides might be based on social norms, where some reservations are expected to push points through direct talk instead of through intermediate people or directly to the women. With this arrangement, the middle person can choose suggestions, which influences what the ego hears |

| | |
|---|---|
| | from the child caregiver. In another scenario, the two sides might tell the child-caregiver and the contradictory women suggestions.<br><br>While constraint measures the extent to which relationships in the household are redundant, hierarchy describes how the redundancy can be traced to a single household member. In the cases of constraint, alters are directly connected and might discuss what they share with the caregivers; hence, they might not have the freedom to choose child feeding suggestions. |
